# Clonal Hematopoiesis in Liquid Biopsy: From Biological Noise to Valuable Clinical Implications

**DOI:** 10.3390/cancers12082277

**Published:** 2020-08-14

**Authors:** Hiu Ting Chan, Yoon Ming Chin, Yusuke Nakamura, Siew-Kee Low

**Affiliations:** 1 Cancer Precision Medicine Center, Japanese Foundation for Cancer Research, Tokyo 135-8550, Japan; hiuting.chan@jfcr.or.jp (H.T.C.); yoonming.chin@jfcr.or.jp (Y.M.C.); yusuke.nakamura@jfcr.or.jp (Y.N.); 2 Cancer Precision Medicine, Inc., Kawasaki 213-0012, Japan

**Keywords:** liquid biopsy, circulating tumor DNA, clonal hematopoiesis, next-generation sequencing

## Abstract

The use of blood liquid biopsy is being gradually incorporated into the clinical setting of cancer management. The minimally invasive nature of the usage of cell-free DNA (cfDNA) and its ability to capture the molecular alterations of tumors are great advantages for their clinical applications. However, somatic mosaicism in plasma remains an immense challenge for accurate interpretation of liquid biopsy results. Clonal hematopoiesis (CH) is part of the normal process of aging with the accumulation of somatic mutations and clonal expansion of hematopoietic stem cells. The detection of these non-tumor derived CH-mutations has been repeatedly reported as a source of biological background noise of blood liquid biopsy. Incorrect classification of CH mutations as tumor-derived mutations could lead to inappropriate therapeutic management. CH has also been associated with an increased risk of developing cardiovascular disease and hematological malignancies. Cancer patients, who are CH carriers, are more prone to develop therapy-related myeloid neoplasms after chemotherapy than non-carriers. The detection of CH mutations from plasma cfDNA analysis should be cautiously evaluated for their potential pathological relevance. Although CH mutations are currently considered as “false-positives” in cfDNA analysis, future studies should evaluate their clinical significance in healthy individuals and cancer patients.

## 1. Introduction

In recent years, liquid biopsy, which involves genomic profiling of tumors using circulating biomarkers in the bodily fluid, has attracted tremendous interest in the field of cancer diagnosis and management [1]. The accessibility and low invasiveness of blood sampling compared to tumor tissue biopsy received great interest for their potential uses in various clinical applications. Liquid biopsy comprises several circulating tumor circulomes, including, circulating tumor proteins, circulating tumor cells, circulating tumor nucleic acid (DNA and RNA), extracellular vesicles, and tumor-educated platelets [2]. Recent advancement in sequencing technology and bioinformatics allowed accurate detection of genetic alterations in circulating tumor DNA (ctDNA) from the blood. ctDNA is highly degraded DNA fragments released from tumor cells and they recapitulate the tumor’s molecular alterations [3]. However, there are also limitations to this approach. Owing to the complex nature of blood plasma, alterations detected from cell-free DNA (cfDNA) could be tumor-derived or non-tumor derived. Maintaining the sensitivity and specificity to detect true tumor-derived cfDNA from plasma remains the biggest challenge for its routine use in clinical practice. The majority of cfDNA present in the blood are derived from hematopoietic cells [4].

Clonal hematopoiesis (CH), a process that involves the accumulation of somatic mutations in hematopoietic stem cells which leads to clonal expansion of mutations in blood cells, may account for the non-tumor derived mutations detected from plasma. CH is part of the normal process of aging, and they are highly prevalent in the general population [5,6]. These mutations from hematopoietic cells which could disguise as tumor-derived, often present as a source of biological background noise to cfDNA analysis. Incorrect classification of mutations detected in cfDNA analysis as tumor-associated mutations could lead to inappropriate therapeutic decisions for patient management. Furthermore, CH mutations have been reported with several points of clinical significance. Healthy individuals who are carriers of CH have shown an increased risk for developing cardiovascular diseases and hematological malignancies while cancer patients with CH mutations are more likely to develop therapy-related myeloid neoplasms several years after the completion of chemotherapy [5,7]. These observed pathological associations highlight the potential clinical importance of CH detected from liquid biopsy, therefore, CH should not be merely perceived as a source of biological background noise to cfDNA analysis. In this review, we have summarized the current understandings of CH as a form of somatic mosaicism in blood liquid biopsy and the reported clinical importance of CH in both healthy individuals and cancer patients. Lastly, we discussed the future directions and perspectives of the valuable clinical implications of CH detected in liquid biopsy.

## 2. Circulating Cell-Free DNA (cfDNA) and Circulating Tumor DNA (ctDNA) in Liquid Biopsy

The concept of cfDNA in human blood was first introduced to the scientific community around 70 years ago [8]. The origin and biology of cfDNA had been extensively discussed in previous reviews [9,10,11,12]. In brief, cfDNAs are highly degraded DNA fragments released from apoptosis, necrosis, and secretion from cells [13,14]. cfDNA circulates in the blood as nucleosomes with a model fragment length of 167 bps [15,16]. Recent studies have shown a distinct nuclear fragmentation pattern with variable fragment lengths of cfDNA from different tissue of origin [17,18,19,20]. The reported half-life of cfDNA in the blood circulation ranges from 4 min to 2 h depending on the physiological state and pathological condition of the individual [21]. It has been shown that the majority of cfDNA in plasma of healthy individuals originate from hematopoietic cells: 55% from white blood cells, 30% from erythroid progenitors, 10% from endothelial and 1% from hepatocytes [4,22,23].

The presence of cfDNA that originates from tumor cells, also known as ctDNA, in the plasma of cancer patients has been well documented from the early-1990s [24,25,26,27]. The minimally invasive nature of cfDNA sampling allows serial sampling and real-time monitoring of cancer patients. Additionally, liquid biopsies may allow the capturing of ctDNA released from multiple tumor regions and could reflect intratumoral-heterogeneity that might be missed in tissue biopsy [28,29]. The development of next-generation sequencing (NGS) has assisted the validation of clinical applications of ctDNA in cancer management, including, tumor profiling [30,31], early cancer detection [32,33], minimal residual disease detection [34,35] and treatment monitoring [36,37,38].

Despite the advantages of liquid biopsy compared with tissue biopsies, optimizing the sensitivity and specificity of NGS used in ctDNA detection remains as one of the key challenges to its utilization in the clinics. The amount of ctDNA shed into the circulation is dependent on the tumor burden, tumor location, vascularity, and cellular turnover [32,39]. Smaller tumor burdens usually shed less ctDNA into the bloodstream than larger tumor burdens [40]. A recent study developed a mathematical model to predict the shedding rate of early-stage non-small cell lung cancer (NSCLC) [41]. From this study, it has been estimated that there would be an average of only 1.7 genome copies of ctDNA in 15 mL of blood for lung tumors with a volume of 1 cm^3^. This highlights the minute amount of ctDNA present in plasma in early-stage cancer. The use of molecular barcoding in ultra-deep sequencing of cfDNA has now become a standard approach to reduce errors that are often observed in conventional NGS sequencing and to increase the detection of low copies of ctDNA present in early-stage cancer. Plasma cfDNA is a complex mixture of mutations derived from germline DNA and malignancy [42]. Even though tumor-derived fragments possess specific characteristics, such as shorter fragment size compared to cfDNA of non-tumor origin [15,43,44,45], the biological mosaicism complicates and limits the specificity of true ctDNA identification from plasma. Furthermore, since the majority of cfDNA arise from hematopoietic cells, mutations originating from non-malignant hematopoietic cells present as an additional natural biological confounding factor.

## 3. Clonal Hematopoiesis

### 3.1. Definition of Clonal Hematopoiesis (CH)

Studies of X-chromosome inactivation in the early 1990s led to the discovery that clonal expansion of blood cells was not only occurring in hematological malignancies but also in healthy individuals as a result of aging [46,47]. It has been estimated that each hematopoietic stem cell acquires one exonic mutation per decade of a normal healthy individual’s life. Based on the estimation that an adult human has an approximately 50,000 to 200,000 stem cells [48], an average person would potentially harbor up to 1.2 million exonic mutations by the age of 70 [6]. The hematopoietic cells with naturally occurring mutations may have the advantage of expanding more rapidly than non-mutated cells; this process is also known as CH [6]. Many reputable studies have been conducted in the past decade to explore CH and different terminologies and definitions were used. It is now generally accepted that the term “CH” refers to any clonal outgrowth of hematopoietic cells, regardless of cause or disease state, while clonal hematopoiesis of indeterminate potential or CHIP usually refers to mutations in driver genes known to be associated with hematological malignancies. CHIP is detected in the DNA of white blood cells from individuals without any symptoms or clinical presentations of malignancy [49]. These mutations will also need to be detected with a minimum variant allele frequency (VAF) of 2% to be classified as CHIP [49]. The terms CH and CHIP will also be used with the definitions mentioned above throughout this review.

### 3.2. The Prevalence and Mutation Profile of CH

Since 2014, several large cohort studies were conducted to examine the mutation profile of CH-related mutations and their prevalence in individuals without known hematological malignancies [5,50,51,52,53,54,55,56,57,58] (Table 1). The majority of the studies included more than thousands of subjects. The reported prevalence of CH varied across studies; however, a notable increase in the detection of CH with increasing age was consistently observed. The most commonly detected CH mutations are from genes *DNMT3A*, *TET2* and *ASXL1*, which are epigenetic modulators [57,59,60,61,62], followed by *JAK2*, *PPM1D*, *TP53*, *IDH2*, *SF3B1* and *SRSF2* [5,50,52,53,57]. These genes are also commonly mutated in hematological malignancies, such as acute myeloid leukemia (AML) and myelodysplastic syndrome (MDS). Besides the hematological-related mutations, there is increasing evidence of CH mutations detected in genes that are commonly mutated in solid tumors, including *KRAS*, *GNAS*, *NRAS*, and *PIK3CA* [52,53,63,64].

**Figure 1:**
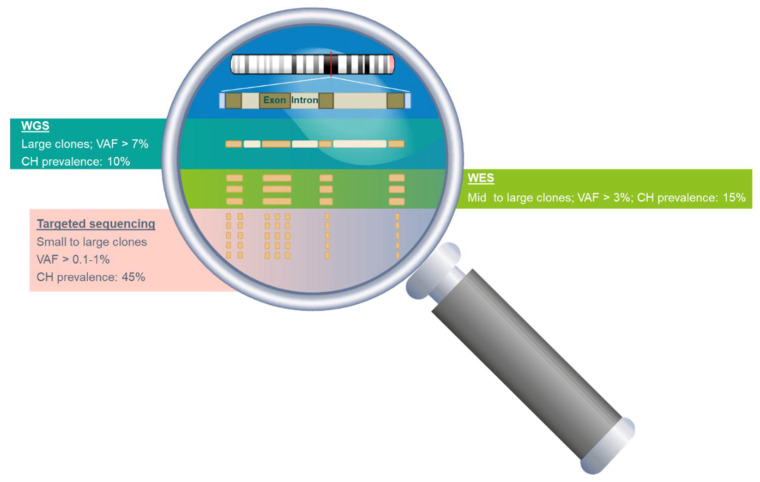
Comparison of the limit of detection and the reported CH prevalence at age 70 by different sequencing methods. Whole-genome sequencing (WGS), sequences the entire genome at a low sequencing depth resulting in a limit of detection of 7%. Whole-exome sequencing (WES) only detects coding variants but can achieve a greater depth of sequencing than WGS. Targeted sequencing specifies a selection of genomic locations and it can achieve greater sequencing depth with a lower limit of detection than either WGS or WES.

**Figure 2:**
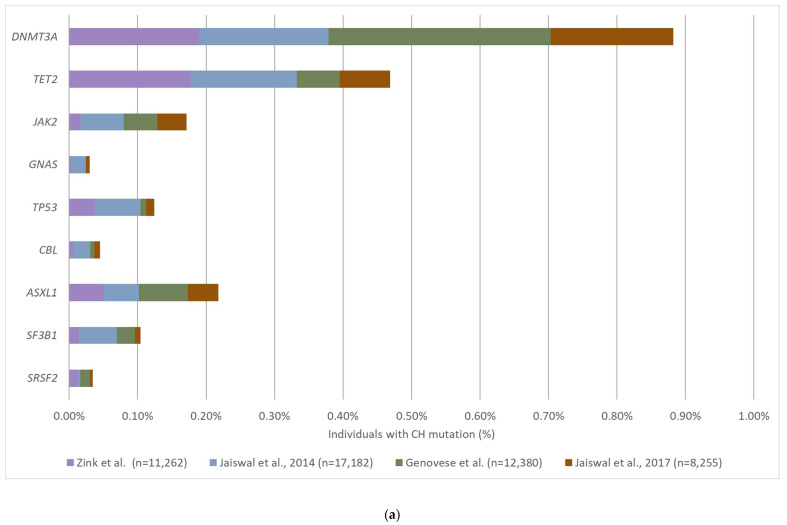

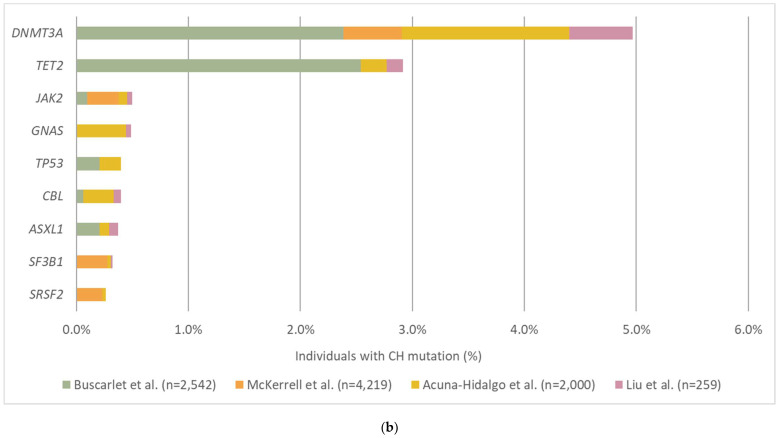
Most frequently altered CH genes detected in healthy individuals. The nine most frequently altered CH genes were selected based on the 4 published studies using targeted sequencing of white blood cells from healthy individuals. The prevalence of each altered gene was calculated by adding the total number of individuals detected with a CH mutation from the gene and divided by the total number of study objects across 4 studies. Studies that did not cover the genes in its panel were excluded from the calculation of the total number of study subjects to avoid bias. The prevalence of the selected CH genes was calculated in the same way for studies that used WGS/WES. (**a**) Prevalence calculated from the WGS/WES studies. (**b**) Prevalence calculated from the targeted sequencing studies.

The variable read depth and the resulting NGS sensitivities in different methodologies may have contributed to the diverse CH detection rate and mutational profile observed in the population (Figure 1). Using NGS methods with low sequencing depth, such as whole-exome sequencing (WES), CH mutations were detected in only 1–2% of individuals at the age of 40 and 10–15% in individuals above the age of 70 [5,50]. In contrast, CH is found in 10–50% of individuals in their 40s and 25–75% at the age of 70 years or older using targeted NGS [52,53,56]. Since the size of the clone that can be detected by NGS is inversely proportional to the sequencing depth, the utilization of different sequencing methods may also affect the detection rate of CH mutations. For example, CH mutations in *DNMT3A* were detected in approximately 0.9% of the healthy subjects by WES while the prevalence increases to 5% when targeted sequencing was applied (Figure 2). In contrast to the most commonly mutated genes in CH (*DNMT3A*, *TET2,* and *ASXL1*) which are often detected at VAF of 10–20%, mutations detected from solid-tumor related genes (*KRAS*, *GNAS*, *NRAS,* and *PIK3CA*) are often detected at a much lower VAF (0.1–0.5%) [52,53,63,64]. The high sensitivity of targeted sequencing enables the detection of CH mutations with a small clone size. For instance, the detection rate of *GNAS* mutation is increased 16-fold when using targeted sequencing compared to WGS/WES (0.5% and 0.03% respectively) (Figure 2). Thus, the sequencing method selected for a study should be carefully considered and chosen based on the purpose of the investigation.

### 3.3. Clinical Implications of CH

#### 3.3.1. Hematological Cancer

The presence of CH does not necessarily indicate hematological malignancies or any alterations in the blood cell counts that reflect the clinical presentation of malignancy. However, the association between CHIP and the risk of developing hematological malignancies has been well documented in previous studies [5,50,51,65,66]. In population-based studies that were followed up for several years, there was a 2–13 fold increase in the relative risk of developing hematological malignancies in individuals that harbored CHIP [5,50,51,65]. Although the relative risk of developing hematological malignancies in individuals with CHIP is significant, the absolute risk remained low. It is estimated that approximately 0.5–1% of CHIP cases would progress to malignancy per year [5,50]. However, individuals having CH mutations with higher frequencies (VAF > 10%) were fivefold more likely to develop hematological malignancies compared to those having mutations with lower VAF [5,66]. Large population case-control studies with 10 years of follow-up improved the understanding of the association between CHIP and AML [65,66]; mutations in genes such as *TP53*, *IDH1*, and *IDH2* demonstrated increased specificity and penetrance for the development of AML. However, many of the individuals who carried mutations in these genes also harbored mutations in other driver genes, such as *NPM1* and *FLT3* [5,50,65]. The mutations in these two driver genes are often absent before the development of AML [5,50,65]. These observations suggest that CH itself is likely to be insufficient to induce malignancy and mutations in additional driver genes are required for the development of hematologic malignancy. Further studies are required to evaluate the potential use of CH mutations for risk assessment of hematological malignancies. Since CH mutations originate mostly from hematopoietic stem cells which differentiate to the full spectrum of hematopoietic cells, it is theoretically possible that CH could predispose to any type of hematological malignancies. However, most conducted studies have only observed the association of CH with myeloid malignancies. Future studies with large cohorts should investigate the association of lymphoid neoplasms and CH [67].

#### 3.3.2. Therapy-Related Myeloid Neoplasms (t-MN)

Several studies have suggested that cancer patients who carry CHIP mutations before the initiation of chemotherapy are more likely to develop t-MN compared to those with no CHIP [53]. It is believed that exposure to chemotherapy acts as an external pressure to favor the survival of hematopoietic stem cells with CHIP and has the advantage of outgrowing those cells without CHIP. Notably, an increasing number of CHIP mutations with high VAF are associated with an increased risk of developing t-MN. The mutational spectrum in patients who developed t-MN had a higher prevalence of mutations in DNA damage response genes such as *TP53* [68]. The early acquisition of *TP53* mutations in CH contributes to the poor responses to chemotherapy seen in patients with t-AML/t-MDS. The clones with CH *TP53* mutations are most likely to be resistant to chemotherapy and expand as a result of selective pressure [68]. Future large populational studies are required to expand our current understanding of CH and therapy-related hematological malignancies to accurately predict which individual with CHIP would proceed to develop malignancies.

#### 3.3.3. Cardiovascular Disease (CVD)

The association between CH and human disease is not limited to cancer. Several studies have found that CHIP carriers were 2–4 times more prone to developing coronary heart diseases including myocardial infarction and ischemic stroke than those without CHIP [5,55,69]. In particular, one recent study (the largest so far) conducted exome sequencing of over 35,000 individuals without previous cardiovascular diseases [69]. The study identified individuals with *DNMT3A* and *TET2* CHIP had an increased risk of cardiovascular disease compared to non-carriers after nearly 7 years of follow-up [69]. Furthermore, the authors of this study observed that CHIP carriers with genotypes of reduced IL-6 signaling abrogated the risk of CVD. Similar to hematological malignancies, the greater risk was also observed in individuals who harbor CHIP with VAF greater than 10% [69]. *TET2*, *ASXL1*, *JAK2*, and *DNMT3A* are the most commonly detected CH mutations in CVD patients [55,70]. Particularly, in individuals bearing *JAK2*-V617F, the relative risk of coronary heart disease was 12 times higher compared to non-carriers, which was also much higher compared to mutations in other genes [55]. CHIP carriers with pre-existing CVD have also been shown to have worse survival outcomes and increased disease progression than those without CHIP [70]. The profound effect of CH mutations on the prognosis of CVD was also observed in patients with severe calcified aortic valve stenosis who underwent transcatheter aortic valve implantation [71]. The mortality rate in patients with CH mutations was nearly three times higher compared to the non-carriers [71].

## 4. CH in Liquid Biopsy

### 4.1. Detection of CH from Plasma

A handful of studies have been conducted in the past few years to assess the detection of CH mutations in plasma and their impact on the interpretation of blood liquid biopsy results (Table 2). The hypothesis of the detection of CH mutations in plasma cfDNA was first suggested in two exploratory studies in small-cell lung cancer (SCLC) and NSCLC patients [64,72]. A total of 5–15% of *TP53* mutations detected in the plasma cfDNA of lung cancer patients were also detected in white blood cells, suggesting their CH origin. These early observations were validated in a recent prospective study that performed deep sequencing of cfDNA and matched white blood cells over 124 patients with metastatic cancer using a large gene panel (508 genes) [73]. Close to 50% of the mutations detected in plasma cfDNA were also detected in patient-matched white blood cells. Similar detection rates of CH mutation from plasma cfDNA was also reported in early-stage NSCLC patients [74]. In addition, paired sequencing of plasma cfDNA and white blood cells from healthy individuals showed that the vast majority of mutations (66–90%) detected in plasma cfDNA were originated from CH [56,73,74,75]. The sequencing gene panel, sequencing depth, and the resulting limit of detection varied among these studies, therefore, it is difficult to directly compare the results among the studies. However, the key message is that CH mutations can contribute greatly to the mutations detected from liquid biopsy and clear assessment should be made to identify tumor-derived cfDNA from plasma samples.

**Figure 3:**
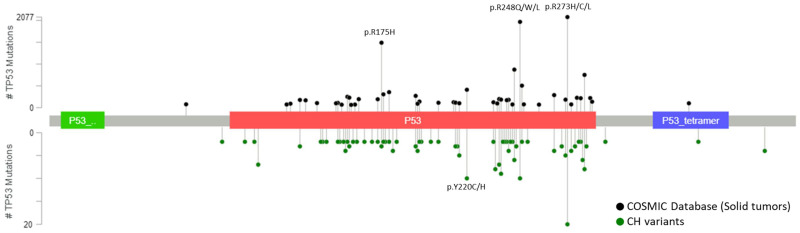
Most frequently detected TP53 variants from solid tumors and white blood cells. Positions and frequencies of most frequently detected *TP53* mutations in solid tumors from COSMIC database (top plot) and in white blood cells from 14 published studies (bottom plot). All reported variants of *TP53* from the COSMIC database were gathered and variants detected from hematopoietic and lymphoid neoplasms were filtered leaving only the variants detected from solid tumors. CH *TP53* variants were similarly gathered from 14 reported studies (References [5,48,49,50,53,56,62,70,71,72,73,74,75,76]). Variants without genomic positions documented were further removed from the analysis leaving a total of 801 CH mutations across 657 different variants. All variants that were reported more than once across the 14 studies were used for the analysis and compared with the top 85 most frequently detected *TP53* variants from solid tumors. Lollipop plots were generated using the MutationMapper from cBioPortal [83,84]

CH mutations detected from plasma are similar to the mutations detected from white blood cells, involved both canonical CH genes, *DNMT3A*, *TET2*, *ASXL1* and *JAK2*, and actionable mutations in genes related to solid tumors, *KRAS*, *PIK3CA* and *EGFR* [56,64,73,75]. Majority of these identified CH variants have been previously reported as tumor-associated somatic mutations which complicate the curation of the variants detected in cfDNA analysis [76,77]. A total of 656 distinct *TP53* variants have been reported as CH mutations in 14 previous studies [5,50,51,52,55,58,64,72,73,74,75,76,77,78] and up to 99% (650/656) of these mutations have been documented in the COSMIC database as somatic mutations detected in solid tumors. Further stratification to focus on the most frequently mutated *TP53* variants in CH showed these hotspot variants coincide with the most frequently mutated *TP53* variants in solid tumors (Figure 3). VAF of CH mutations detected from plasma are highly correlated and indifferent to the VAF detected in white blood cells [73,75] which highlights the importance of sequencing white blood cells to at least the same depth as cfDNA to correctly exclude the CH mutations and avoid misinterpretation. Furthermore, our group has recently shown there are no significant differences between the VAF of CH-related and tumor-derived ctDNA detected in plasma samples [76]. The indifferences in the type of variant and VAF between CH and ctDNA mutations reinforce the difficulties to differentiate them without performing paired deep sequencing of plasma cfDNA and DNA from white blood cells.

Recent studies have focused on increasing the understanding of biophysical and genomic features of CH and ctDNA to assist the classification of cfDNA mutations. Several proof-of-concept studies indicated that ctDNA presents as shorter fragment size distribution than CH or non-mutated cfDNA fragments [74,77,79]. The unique shorter fragment size of ctDNA could help to identify tumor-derived cfDNA mutations. Furthermore, the majority of point mutations observed in CH are C>T transitions, which are derived from the spontaneous deamination of methylated cytosine into thymine, also consistent with the aging-related mutational signature (signature 1) [5,53,58,80,81]. Recent studies have found this age-related mutational signature to be enriched in CH cfDNA fragments and absent in ctDNA fragments [56,74]. In contrast, smoking mutational signature (signature 4) was exclusive to tumor-derived fragments and absent in CH cfDNA in NSCLC patients [74]. These observations suggest that biophysical and genomic features of cfDNA variants might be useful for distinguishing tumor-derived mutations from CH.

### 4.2. Detection of CH from Tumor Tissues

Tumor-informed liquid biopsy analysis has become one of the common approaches used by research groups to overcome the problem of somatic mosaicism in plasma [35,74,85,86,87]. It involves sequencing the tumor tissue (surgically resected or tissue biopsy) to identify and select tumor-specific mutations that are to be further monitored using plasma cfDNA for various clinical applications. The theory that underlies this approach is that all mutations detected from tumor tissues would be specific to the malignancy. However, such an approach should also be carefully considered as CH mutations may also be present in tumor tissues due to tumor-infiltrating blood cells [53,63,76]. Two large retrospective studies analyzed existing NGS data of paired tumor tissues and white blood cells from thousands of cancer patients with various solid tumors to assess the prevalence of CH mutations in tumor tissues [53,63]. A total of 14–77% of CH mutations detected in white blood cells were detected in tumor tissues. The VAF of CH mutations detected in tumor tissues ranged from 0.5 to 21% [53,63]. As mentioned previously in this review (Figure 1), the limit of detection for an NGS assay is directly correlated to the sequencing depth. The VAF of CH in tumor tissues could go below 0.5% with increasing sequencing depth and their prevalence could be higher than previously reported [76]. These results highlight the importance of white blood cell sequencing even for tumor-informed analysis to exclude the possibility of CH. The clinical implications of these findings are not limited to liquid biopsy, CH mutations should also be considered when performing tumor profiling using tumor tissues to prevent incorrect identification of targetable alterations.

### 4.3. Clinical Impact of CH Mutations on the Interpretation of Liquid Biopsy Results

The presence of CH in cfDNA of the general population has now been well established, however, limited studies have been conducted to directly examine the impact of CH on the clinical interpretation of blood liquid biopsy results. cfDNA analysis has been suggested as a promising tool for cancer screening. As mentioned in previous studies, a large proportion of mutations detected in plasma cfDNA of healthy individuals could be originated from CH [56,73,75]. Misinterpretation of these CH-related mutations as ctDNA mutations may lead to unreliable diagnosis.

Liquid biopsy was recently approved to identify actionable alterations in specific genes that could assist in treatment selection when tumor tissue is unavailable. CH mutations should be carefully considered for these assays as recent studies have shown that a substantial number of CH variants are considered to be oncogenic and are indicated for molecular-targeted therapies [64,73,77]. In the study conducted by Razavi et al., up to 10% of the CH mutations detected in plasma were listed as oncogenic in OncoKb database and 13% of these mutations were indicated for either an approved targeted therapy or a treatment under clinical trial. Incorrect identification of actionable alteration would lead to inappropriate treatment.

The minimal invasiveness of liquid biopsy and the ability for serial sampling highlight its usefulness for the detection of minimal residual disease and monitoring of treatment response for solid tumor patients [34,35,88,89,90]. However, most of the studies conducted earlier did not exclude CH mutations in the analysis. We evaluated the impact of misclassification of CH as ctDNA on the clinical interpretation of cfDNA results in one of our recent studies [76]. In our colorectal cancer patients study cohort, 17% of the pre-operative cfDNA mutations were identified to be CH-related. The recruited patients were followed post-operatively and the identified CH mutations were recurrently detected after surgery or completion of adjuvant chemotherapy. Under unpaired sequencing of cfDNA, the consistent detection of CH could be incorrectly interpreted as the presence of residual disease after tumor resection or inappropriately inferred as disease progression or treatment ineffectiveness.

### 4.4. Other Potential Sources of Non-Tumoral Somatic Mutations

Results from others and our study have shown that, besides CH from white blood cells and tumor-derived mutations, there are still other cfDNA mutations detected in the plasma with an unknown origin [73,76]. Future studies are needed to investigate whether hepatocytes and endothelial cells may also contribute to cfDNA mutations detected in plasma [4,22,23]. Furthermore, in healthy subjects, DNA from the erythroid lineage may contribute up to 30% of plasma cfDNA [22,23]. It has been suggested that CH may also affect the erythroid lineage and carries unique somatic mutations that are different from white blood cells [91]. Although mature red blood cells do not have a nucleus, erythroblasts lose their nuclei and are matured into reticulocytes in the bone marrow during the enucleation step [23]. The nuclear material of the erythroblasts gets degraded and may be released into the circulation as a form of cfDNA, therefore, it is of great interest to investigate whether cells in the erythroid lineage are another contributor to the cfDNA mutations detected in plasma.

## 5. Future Directions: Clinical Interpretation of CH

The rapid development and validation of ctDNA-based liquid biopsy in observational studies and clinical trials has allowed several ctDNA-guided therapies to be approved for their use in cancer patients. Furthermore, the full clinical utilities of ctDNA in oncological management are also currently being explored through large-scale interventional clinical trials [92]. However, cfDNA analysis should be further optimized to support the use of blood liquid biopsy as a routine practice with a clinically affordable cost in cancer management.

The current approach of performing paired cfDNA-white blood cells DNA sequencing to differentiate CH mutations doubles the costs involved which may reduce the cost-effectiveness of ctDNA analysis. In the study conducted by Chabon et al., the authors utilized machine learning to incorporate molecular and genomic features of CH and tumor-derived cfDNA fragments to identify the sources of cfDNA mutations [74]. Integration of biophysical, genomic, and molecular features of CH and ctDNA together with large datasets of CH mutations and tumor mutation profile may refine the machine learning model to assist the identification of ctDNA mutations.

Our understanding of the contribution of CH to the mutations detected by cfDNA analysis has improved tremendously in the past decade. However, there are still many unknowns and unanswered questions that need to be addressed to maximize the potential utilities of blood liquid biopsy. CH-related mutations detected from liquid biopsy may provide important information for assessing hematologic malignancy and CVD risks in healthy individuals. Although the association of CH and its clinical implications has been well documented, a clear VAF cut-off to determine the relative or absolute risk of developing diseases should be further investigated. Current detection of CH mutations is often unintentional; further investigation is needed to assess whether monitoring healthy individuals who are CHIP carriers would bring any clinical benefits by allowing earlier detection of hematological malignancy or CVD. Similarly, in the field of oncology, cancer patients who are CHIP carriers are more prone to develop t-MN after chemotherapy. Future studies should assess the overall clinical benefits of chemotherapy in CHIP carriers with long-term monitoring.

## 6. Conclusions

As the use of plasma cfDNA analysis in the clinical setting continues to grow, extra caution should be taken to accurately determine the origin of the mutations and differentiate tumor-derived ctDNA from the biological confounding factors present in the blood, such as CH mutations. Incorrect interpretation of liquid biopsy results can directly affect diagnosis and compromise clinical management of cancer patients. The current approach to identify CH is by performing paired sequencing of plasma cfDNA and DNA from white blood cells. However, the extra costs involved in paired sequencing may become an impeding factor for the routine use of liquid biopsy in clinical practice. A better understanding of the biophysical, molecular, and genomic features of CH with the integration of machine learning may reduce the need to perform white blood cells-paired sequencing. The practice of performing white blood cell sequencing in cfDNA analysis has increased the detection rates of CH in the general population. However, there are currently no guidelines to address the detection of these mutations. Future studies should validate the clinical implications of CH detected in cfDNA analysis to optimize the utilities of liquid biopsy.

## Figures and Tables

**Table 1:** Summary of published studies on the prevalence of clonal hematopoiesis (CH) mutations in individuals without known hematological malignancies.

Sequencing Method	Study Size	Participants	Age Range	Depth *	Reported LOD (%)	CH Prevalence	Study
WGS	11,262	Icelanders with various diseases	10–100	36x	10%	12%	Zink et al., 2017 [51]
WES	17,182	Healthy controls	19–108	84x	3.5%	Age 70–79: 9.5% Age 80–89: 11.7% Age 90–108: 18.4%	Jaiswal et al., 2014 [5]
WES	12,380	Healthy controls	19–93	NR	5%	Age < 50: 1% Age > 65: 10%	Genovese et al., 2014 [50]
WES	2728	Patients with solid tumors	10–90	108x	3%	Age > 70: 5%	Xie et al., 2014 [58]
WES	8255	Patients with cardiovascular disease and healthy controls	Median: 60	NR	3%	CVD: 17% Controls: 10%	Jaiswal et al., 2017 [55]
Targeted NGS (15 hotspot mutations)	4219	Healthy controls	17–98	1000x	0.8%	Age < 60: 0.8% Age ≥ 90: 19.5%	McKerrell et al., 2015 [54]
smMIPs	2000	Healthy controls	20–69	845x	0.1%	Age 60–69: >20%	Acuna-Hidalgo et al., 2017 [52]
Targeted NGS	8810	Patients with solid tumors	1–98	419x	1%	25%	Coombs et al., 2017 [53]
Targeted NGS (19 genes)	2530	Women without a known hematological disorder	55–101	4000x	0.1%	13.7%	Buscarlet et al., 2017 [57]
Targeted NGS (559 genes)	259	Healthy controls	Median: 47	6200x (Collapsed: 680x)	0.1%	Age > 50: 76%	Liu et al., 2019 [56]

* Refers to raw sequencing depth unless otherwise stated; NR: not reported; LOD: limit of detection; WGS: whole-genome sequencing; WES: whole-exome sequencing; CVD: cardiovascular disease, smMIPs: single-molecule molecular inversion probes; NGS: next-generation sequencing.

**Table 2:** Summary of published studies on the detection of CH mutations from plasma cell-free DNA (cfDNA) analysis.

Cancer Type	Stage	Study Size	Gene Panel	Depth *	Reported LOD (%)	Prevalence of CH Detection from Plasma cfDNA Analysis	Study
**SCLC**	I–IV	SCLC: 51 Healthy controls: 123	*TP53*	NR	NR	SCLC: 5.3% Controls: 15%	Fernandez-Cuesta et al., 2016 [72]
**Cancer-free**	-	821	50 genes	40,000x	0.10%	89%	Xia et al., 2017 [75]
**NSCLC**	III–IV	122	Focused on *TP53* analysis	NR	NR	15%	Hu et al., 2018 [64]
**Prostate**	IV	217	305 genes	814x	1%	15%	Mayrhofer et al., 2018 [78]
**Cancer-free**	-	259	599 genes	cfDNA: 6200x white blood cells: 406x	0.25%	66%	Liu et al., 2019 [56]
**Various solid tumors**	IV	Cancer:124 Healthy controls: 47	508 genes	60,000x (collapsed: 4500x)	0.1%	Cancer: 53% Controls: 82%	Razavi et al., 2019 [73]
**Gastric**	I–IV	788	58 genes	30,000x	0.1%	44%	Leal et al., 2020 [77]
**Renal cell carcinoma**	IV	55	981 genes	Collapsed: 938x	1%	20%	Bacon et al., 2020 [82]
**NSCLC**	I–III	NSCLC: 104 Healthy controls: 98	255 genes	Collapsed: 4000–5000x	0.01%	NSCLC: 58% Controls: 90%	Chabon et al., 2020 [74]
**CRC**	I–IV	38	52 genes	48,000x (collapsed: 4000x)	0.1%	17%	Chan et al., 2020 [76]

* Refers to raw sequencing depth unless otherwise stated; NR: not reported, SCLC- small cell lung cancer; NSCLC- non-small cell lung cancer; CRC- colorectal cancer; LOD- limit of detection.
